# Supplementary material for: Reliability and Concurrent and Construct Validity of a Food Frequency Questionnaire for Pregnant Women at High Risk to Develop Fetal Growth Restriction
Source: Nutrients. 2021 May 12;13(5):1629. doi: 10.3390/nu13051629 (PMC8150790; doi:10.3390/nu13051629)
Supplement: Supplementary file 1 [file nutrients-13-01629-s001.zip › Table S1.pdf]

Table S1. Daily nutrient intake derived by 24h recalls according to adherence to the Mediterranean diet calculated by the food frequency questionnaire.

	MedDiet score (FFQ)				
**Nutrients (24hR)**	**1st quartile** *N=37*	**2nd quartile** *N=38*	**3rd quartile** *N=37*	**4th quartile** *N=38*	**P for trend**
Carbohydrates (g/4.18MJ)	105 [102;109]	104 [99.7;108]	101 [97.5;105]	107 [102;111]	0.778
Proteins (g/4.18MJ)	44.1 [40.5;48.4]	42.4 [41.0;47.7]	43.3 [39.6;47.9]	43.3 [39.1;44.6]	0.522
Lipids (g/4.18MJ)	46.4 [45.1;47.8]	46.6 [45.2;48.0]	47.5 [46.1;48.9]	46.4 [44.7;48.1]	0.768
Saturated fatty acids (g/4.18MJ)	13.5 [12.8;14.3]	13.2 [12.5;13.9]	13.2 [12.5;13.9]	12.5 [11.7;13.3]	0.052
Monounsaturated fatty acids (g/4.18MJ)	21.3 [20.3;22.5]	20.9 [20.0;22.3]	21.5 [20.3;22.3]	21.5 [20.1;22.3]	0.997
Polyunsaturated fatty acids (g/4.18MJ)	6.57 [5.87;7.33]	7.16 [6.43;7.94]	6.73 [6.28;7.49]	7.02 [6.62;8.01]	0.016
Cholesterol (mg/4.18MJ)	156 [134;170]	148 [134;170]	151 [133;167]	140 [132;156]	0.218
Fiber (g/4.18MJ)	10.6 [9.65;12.1]	10.1 [9.09;11.2]	10.6 [10.2;11.8]	11.7 [10.7;12.8]	0.043
Vitamin A (mcg/4.18MJ)	439 [221;614]	535 [416;638]	499 [392;602]	637 [504;829]	0.029
Beta-carotenoids (mcg/4.18MJ)	1230 [609;1787]	1408 [1133;1765]	1419 [1112;1610]	1685 [1384;2148]	0.044
Vitamin B1 (mg/4.18MJ)	0.61 [0.57;0.64]	0.64 [0.61;0.67]	0.62 [0.60;0.66]	0.64 [0.58;0.67]	0.633
Vitamin B2 (mg/4.18MJ)	0.75 [0.72;0.85]	0.78 [0.74;0.84]	0.73 [0.68;0.77]	0.74 [0.68;0.79]	0.037
Vitamin C (mg/4.18MJ)	58.8 [45.9;64.4]	50.4 [41.3;70.3]	59.0 [46.7;69.3]	69.7 [58.7;85.3]	0.015
Vitamin E (mg/4.18MJ)	4.50 [4.00;4.75]	4.62 [4.18;4.81]	4.39 [4.28;4.73]	5.03 [4.48;5.46]	0.016
Zinc (mg/4.18MJ)	6.33 [6.10;7.49]	6.41 [5.87;7.23]	6.52 [6.10;7.05]	6.50 [6.21;7.40]	0.038
Magnesium (mg/4.18MJ)	124 [114;133]	131 [116;137]	121 [115;132]	130 [122;143]	0.554
Potassium (mg/4.18MJ)	1245 [1190;1454]	1320 [1201;1431]	1258 [1214;1432]	1387 [1290;1539]	0.064
